# Reliability and agreement of root length measurements during orthodontic treatment in images from different CBCT machines using multiplanar reconstruction

**DOI:** 10.2340/biid.v11.41161

**Published:** 2024-08-22

**Authors:** Kristina Johansson, Liselotte Paulsson, Helena Christell

**Affiliations:** a Faculty of Odontology, Malmö University, Malmö, Sweden; b Department of Orthodontics, Östersund Hospital, Östersund, Sweden; c Department of Diagnostics, Helsingborg Hospital, Helsingborg, Sweden

**Keywords:** Cone-beam computed tomography, root resorption, orthodontic appliances, fixed, reproducibility of results, observer variation

## Abstract

**Objectives:**

To assess inter- and intrarater reliability and agreement for measurements of root lengths using multiplanar reconstruction (MPR) in cone beam computed tomography (CBCT) examinations.

Furthermore, to determine whether using MPR from different CBCT machines was a reliable and reproducible method for assessment of root length during orthodontic treatment of adolescents.

**Materials and methods:**

A total of 40 CBCT examinations obtained before, during and after orthodontic treatment of 14 adolescents, with fixed appliances from a multicentre randomised controlled trial, were used. All roots from the incisors to the first molars were measured by two independent raters and in accordance with a protocol preceded by a multi-step calibration. Reliability was assessed by intra class correlation (ICC). Agreement was assessed by measurement error according to the Dahlberg formula and Bland–Altman plot.

**Results:**

The number of repeated measurements varied from 436 to 474 for the different timepoints. Good to excellent inter- and intrarater reliability for different tooth groups and timepoints were shown. Measurement error for inter- and intrarater agreement varied between 0.41 mm and 0.77 mm. The Bland–Altman plot with 95% limits of agreement varied between +1.43 mm and −2.01 mm for different tooth groups and timepoints.

**Conclusions:**

The results of this study indicate that CBCT using MPR from different machines is a reproducible method for measuring root length during different phases of orthodontic treatment. When interpreting root shortening measurements in CBCT using MPR for clinical or research purposes, values below 2 mm should be approached with caution, as they may contain measurement errors.

## Introduction

Healthcare interventions come with varying degree of risk for adverse effects [1] whereof orthodontic treatment can involve external apical root resorption (EARR) [2, 3]. The result of EARR is a permanent shortening of the tooth root, with higher frequency and severity noted in the maxilla, particularly in the anterior teeth [4, 5]. Maxillary incisors are most frequently affected, with mean values ranging from 0.42 to 1.30 mm [4], although the individual variation can be substantial and root shortening exceeding 8 mm for some roots has been noted [6]. In general, studies report frequencies of severe resorption (≥ 2 mm) in maxillary incisors ranging from 10 to 29% [7]. EARR can be assessed by measuring the root length in radiographic images before and after orthodontic treatment. Additionally, an intermediate radiographic control 6 to 12 months post treatment start is recommended to determine if any specific action is required to prevent severe EARR [8, 9].

For measurements to be considered valid, low measurement error reflecting a high reliability is essential. Furthermore, the root shortening must exceed the measurement error [10, 11] as all physical measurements include a certain degree of error [12]. Inter- as well as intrarater reliability should be reported to minimise risk of bias in EARR evaluation, as outlined in the Guidelines for Reporting Reliability and Agreement Studies (GRRAS). This also applies to measurements reported in clinical trials [11].

Systematic reviews have consistently demonstrated that cone beam computed tomography (CBCT) surpasses intraoral and panoramic radiography when assessing EARR for research purpose [3, 13]. However, a recent systematic review focusing on in vivo EARR studies related to fixed appliance treatment highlighted a deficiency in reliability and agreement reporting regarding measurements in CBCT [7]. Only one study within this review addressed both reliability and agreement of CBCT measurements. This was also the only study using multiplanar reconstruction (MPR) to orient the tooth root in axial, coronal and sagittal planes in CBCT volumes instead of using preselected CBCT-slices [14]. The resulting measurement error was low, although it varied for different timepoints; but the measurements were performed by only one rater. Conversely, another study with several raters included reported low inter- and intrarater reliability of measurements in CT volumes across three perpendicular planes, which was believed to be due to the raters selecting different CT slices for their assessments [15].

To the best of our knowledge, no earlier study has evaluated reliability with more than one rater measuring root length in CBCT using MPR. The necessity for such studies is underscored by systematic reviews [3, 16, 17], particularly in the evaluation of EARR at different phases of orthodontic treatment.

This study aims to determine the inter- and intrarater reliability and agreement for measurements of root length of all teeth using MPR in CBCT examinations during different phases of orthodontic treatment in adolescents with fixed appliances.

The hypothesis was that the measurements can be performed with good interrater and intrarater reliability, based on results from earlier research using pre-selected slices and MPR. Further, a higher measurement error for agreement compared to earlier studies using pre-selected slices was hypothesised.

The research is part of a multicentre study following a randomised controlled clinical trial (RCT) protocol.

## Materials and methods

The trial design is inspired by a tool developed for systematic reviews of adverse effects [7], and the reporting follows the GRRAS guidelines [11]. The protocol for the clinical trial was registered in clinicaltrials.gov with registration number NCT05664282.

### Trial design, patients and ethics

CBCT examinations from 14 patients were included in this study. Mean age at baseline was 14.43 years (standard deviation [SD] 1.64, range 10.91 to 16.30); 8 patients were male and 6 were female. The sample was selected from CBCT examinations obtained for a clinical trial of fixed orthodontic appliance and no additional radiographs were performed for this study. The included 14 patients were randomly selected by computer generation from patients that had completed CBCT examinations at all the three different per protocol occasions in an ongoing multi-centre two-arm parallel-arm-group RCT with a 1:1 allocation ratio. Inclusion criteria were adolescents with crowding and displaced teeth, treated non-extraction with either passive self-ligating (Damon Q™ 0.022 variable torque, Ormco Corporation, Orange, California, USA) or conventional (Victory low profile APC plus™ 0.022 MBT standard torque, 3M St Paul, Minnesota, USA) fixed appliance systems. The patients were recruited in three orthodontic clinics in Sweden: a university clinic, a private practice, and a specialist clinic in regional care. Before inclusion in the study, informed consent was obtained from the patients and their legal guardians. The trial protocol and informed consent form were approved by the Regional Ethical Review Board in Lund (Dnr. 2014/647), following the Declaration of Helsinki and the local radiation ethics committee in Skåne and Dalarna.

### CBCT examinations

Five CBCT machines at three radiological clinics were used throughout the trial (Table 1), as the current study is part of a multicentre study. Before the CBCT examinations were performed, objective image quality was assessed using the SEDENTEX CT Quality Control (QC) Phantom (Leeds Test Objects Ltd, North Yorkshire, UK). The imaging performance characteristics were used to decide the lowest possible radiation dose related to the diagnostic task according to the As Low As Diagnostically Acceptable (ALADA) principle [18].

**Table 1:** The radiological equipment and parameters used for CBCT examination.

Radiological clinic	CBCT machine	Voltage (kV)	Tube current (mA)	Rotation (degrees)	Scan time (s)	FOV (cm)	Voxel size (μm)	Software	Monitor
A	3D Accuitomo® 170(Morita®, Kyoto, Japan)	80	3/6	360	17.5	8 × 8	160	i-Dixel®, software, (Morita, Kyoto, Japan)	Barco View, MFGD, Belgium
A	Veraview epocs®(Morita, Kyoto, Japan)	80/90	2/3/5	180	9.3/9.4	8 × 8	125	i-Dixel® software, (Morita, Kyoto, Japan)	Barco View, MFGD, Belgium
B	i-CAT® 9140(Envista holdings corp. California, US)	120	5	360	4	8 × 16	300	Romexis® software, (Planmeca Helsinki, Finland)	Barco View, MFGD, Belgium
B	Promax® 3D Mid(Planmeca Helsinki, Finland)	90	5	180	12	8 × 8	200	Romexis® software, (Planmeca Helsinki, Finland)	Barco View, MFGD, Belgium
C	3D Accuitomo® 170(Morita, Kyoto, Japan)	80	3	360	17.5	8 × 8	160	PACS, Sectra IDS7®, (Sectra AB, Linköping, Sverige)	Barco View, MDCC-6330, Belgium

CBCT: cone beam computed tomography; kV: kilovolt; mA: milliampere; s: second; FOV: field of view; cm centimetre; μm: micrometre.

Examinations were made so that all teeth from the incisors to the first molars in both jaws were contained in one volume. Details concerning the CBCT examinations are displayed in Table 1. CBCT examinations were performed before treatment start (T0), during treatment at insertion of the first 0.019 × 0.025 stainless steel archwire in conjunction with completed levelling of the teeth (T1), and after completed active treatment (T2).

### Data processing

The CBCT examinations from Clinics A and B were all reviewed at the same workstation at Clinic A. The CBCT examinations from Clinic A could be reviewed directly in iDixel software while the CBCT examinations from Clinic B were stored in Digital Imaging and Communications in Medicine (DICOM) file format and transferred to the workstation at Clinic A for review and measurements in Romexis software. At Clinic C, axial slices were sent to Sectra Picture Archiving and Communication System (PACS) software using DICOM export and were then reformatted using MPR to be reviewed (Table 1).

### Raters, reformatting, and measurements

Measurements were made in CBCT examinations at all three timepoints: T0, T1 and T2, by two raters: an orthodontist and an oral and maxillofacial radiologist with experience of 18 and 5 years, respectively. The raters were not involved in the treatment of patients. All maxillary and mandibular roots from the incisors to the first molars for the 14 patients were measured in the same order each time. The CBCT examinations were coded, but blinding in terms of timing was not possible, as the observers could see if the teeth were crowded (T0), had brackets on (T1) or were aligned (T2).

**Figure 1:**
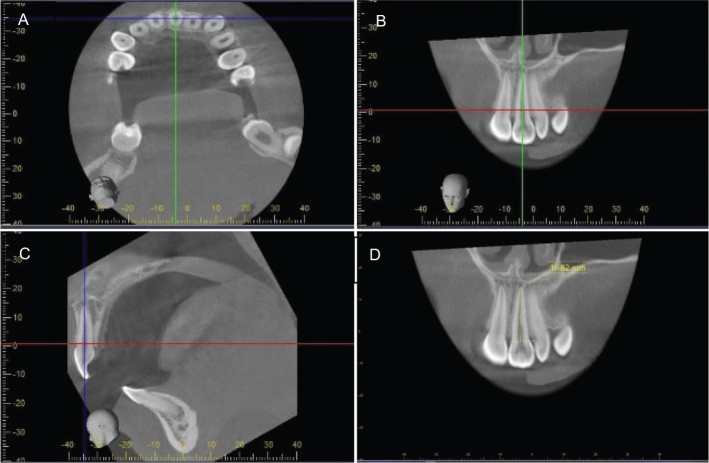
Cone beam computed tomography (CBCT) images in three perpendicular planes with orientation of each tooth using multiplanar reconstruction (MPR). The MPR involved angulation of the tooth in the axial plane according to its rotation in the tooth row (A), and then further angulation for its long axis to be parallel to the axes of the sagittal plane (B) and the coronal plane (C). The measurement of the root was made in the image plane where the tooth was seen buccally (D). Therefore, first molars, premolars and canines were measured in the sagittal plane, while the first and second incisors were measured in the coronal plane. A reference line from the mesial to the distal cemento–enamel junction was marked, and the root was measured from the reference line along the axis of the root to the tooth apex.

A measurement protocol developed by Lund [14] was modified after a calibration process in three steps. Firstly, the protocol was discussed and clarified. Secondly, for the two raters to gain experience using MPR and the protocol, repeated measurements were performed of all maxillary and mandibular roots from incisors to first molars in three CBCT examinations, not included in this study. Thirdly, 20 CBCT examinations from 10 patients included in the clinical study, from timepoints T0 and T2, were rated individually by the two raters. Interrater Intraclass Correlation (ICC) was found insufficient, and therefore a final discussion and adjustment were made, resulting in the finalised protocol as follows:

- Each rater used MPR individually to provide an optimal visualisation of each individual tooth/root in axial, coronal, and sagittal planes (Figure 1).
- Measurements were made in the image-plane showing the buccal aspect of the tooth (Figure 1). Zooming tools and maximisation of windows were used when applicable [19].
- The cemento-enamel junction (CEJ) was marked out with a line from the mesial to the distal root surface (Figure 1). If the CEJ could not be identified in either side of the tooth, an assessment from the side where it could be visualised was made.
- Root length measurements were made to the nearest tenth of a millimetre along the axis of the root from the CEJ line to the apex (Figure 1). If a root was bent, firstly, the distance from the CEJ to the point where the root was bent was measured, and secondly, the distance from that point to the apex was measured. Then the distances were summed up. In teeth where two of the roots had a common apex, only the buccal root was measured. Any root deflections or open apices were noted. All readings were performed in a room with subdued lighting.

All roots from the incisors to the first molars were measured independently, at T0, T1 and T2, once by HC and twice by KJ to assess inter- and intrarater reliability and agreement. All repeated measurements were made with an interval of at least 4 weeks. CBCT examinations were excluded if the image quality was unacceptable. A root was excluded if it was not fully imaged in all three planes or if the image quality was unacceptable, meaning that the tooth apex or the CEJ was not clearly visible in either the mesial or the distal root surface.

### Sample size calculation

In the current study, different statistical analyses were made prompting different sample size calculations. Under the assumption of two raters, a sample size of 40 subjects is recommended to estimate an ICC of 0.9 with a 95% confidence interval (CI) of 0.15 with 80% probability [20]. To provide an estimate of the random error as assessed by Dahlberg´s formula, a minimum sample size of 25–30 is recommended [12]. The analyses in current study were not made per patient but per tooth group (incisors, canines, premolars and molars). To allow for exclusions, and to achieve an appropriate sample size for reliability as well as agreement, a sample size of 56 roots in the tooth group with the lowest number of roots (canines) was determined. To achieve this number of roots, inclusion of 14 patients was needed.

### Statistics

Inter- and intrarater agreement were calculated from duplicate readings using the formula according to Dahlberg [21], and Bland–Altman plots with 95% limits of agreement were performed [22]. Inter- and intrarater reliability were calculated from duplicate readings using ICC with two-way mixed effects [23] and 95% CI. Calculations were made for the roots of the different tooth groups: incisors, canines, premolars and molars. For comparison of inter-and interrater agreement and reliability for the different CBCT machines, calculations were made for all measured roots. The data were analysed using SPSS software (version 25, SPSS, Chicago, Illinois, USA).

## Results

In total, 2,702 reliability and agreement assessments of root measurements in 40 CBCT examinations from the 14 patients were included. One CBCT examination from T0 and T2 respectively and seven tooth roots were excluded due to unacceptable image quality, and one root was excluded as it was not fully imaged in all three planes. The number of roots in different tooth groups varied from 51 to 167 (Table 2).

**Table 2:** Number of repeated measurements of root length – interrater and intrarater – using five CBCT machines at the three radiological clinics.

CBCT machine Radiological clinic		Interrater			Intrarater		
		T0	T1	T2	T0	T1	T2
3D Accuitomo® 170 Clinic A	Incisors	16	-	-	16	-	-
	Canines	8	-	-	8	-	-
	Premolars	21	-	-	21	-	-
	Molars	24	-	-	24	-	-
	Total	69	-	-	69	-	-
Veraview epocs® Clinic A	Incisors	24	36	40	24	35	40
	Canines	12	20	20	12	20	20
	Premolars	32	51	48	32	53	52
	Molars	36	59	60	36	52	52
	Total	104	166	168	104	160	164
iCAT® -9140 Clinic B	Incisors	48	48	40	48	48	40
	Canines	24	24	20	24	24	20
	Premolars	57	58	46	61	57	48
	Molars	72	72	60	72	72	60
	Total	201	202	166	205	201	168
ProMax 3D® Mid Clinic B	Incisors	-	8	8	-	8	8
	Canines	-	4	4	-	4	4
	Premolars	-	12	10	-	12	12
	Molars	-	12	12	-	12	12
	Total	-	36	34	-	36	36
3D Accuitomo® 170 Clinic C	Incisors	16	16	16	16	16	16
	Canines	7	8	8	8	8	8
	Premolars	20	22	20	21	21	22
	Molars	24	24	24	24	24	24
	Total	67	70	68	69	69	70
	Overall Total	441	474	436	447	466	438

CBCT: cone beam computed tomography; T0: time before treatment; T1: time during treatment; T2: time after treatment.

**Figure 2:**
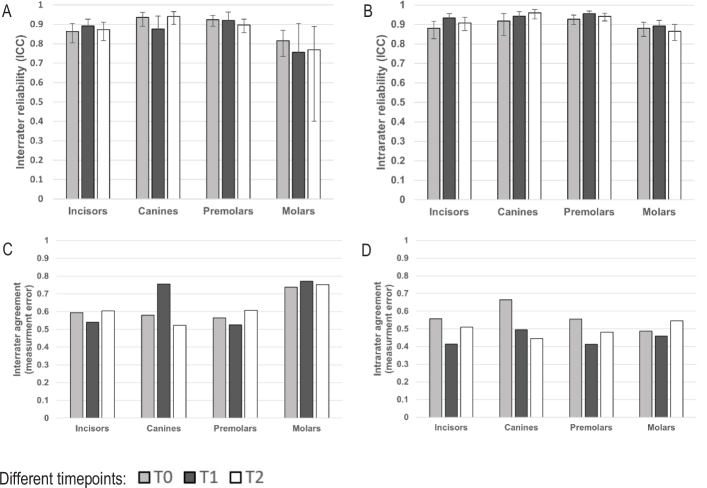
Reliability and agreement of repeated measurements of root length for four different tooth groups (incisors, canines, premolars and molars) in CBCT images. Calculations of reliability by ICC with 95% confidence interval (A, B) and agreement by measurement error in millimetres, according to Dahlberg’s formula (C, D). The results varied between timepoints and tooth groups with ICC for interrater reliability ranging from 0.77 to 0.94 (95% CI: 0.4–0.97) (A), whereas intrarater ICC varied between 0.87 to 0.96 (95% CI: 0.82–0.98) (B). Measurement error for interrater agreement ranged between 0.52 and 0.77 (C), whereas intrarater agreement ranged from 0.41 to 0.66 (D). CBCT: cone beam computed tomography; ICC: intraclass correlation; CI: confidence interval; T0: time before treatment; T1: time during treatment; T2: time after treatment.

The reliability and measurement error varied between timepoints and tooth groups with interrater ICC ranging from 0.77 to 0.94 (95% CI: 0.4–0.97), whereas intrarater ICC varied between 0.87 and 0.96 (95% CI: 0.82–0.98). Measurement error for interrater agreement ranged between 0.52 and 0.77, whereas intrarater agreement ranged from 0.41 to 0.66 (Figure 2).

**Figure 3:**
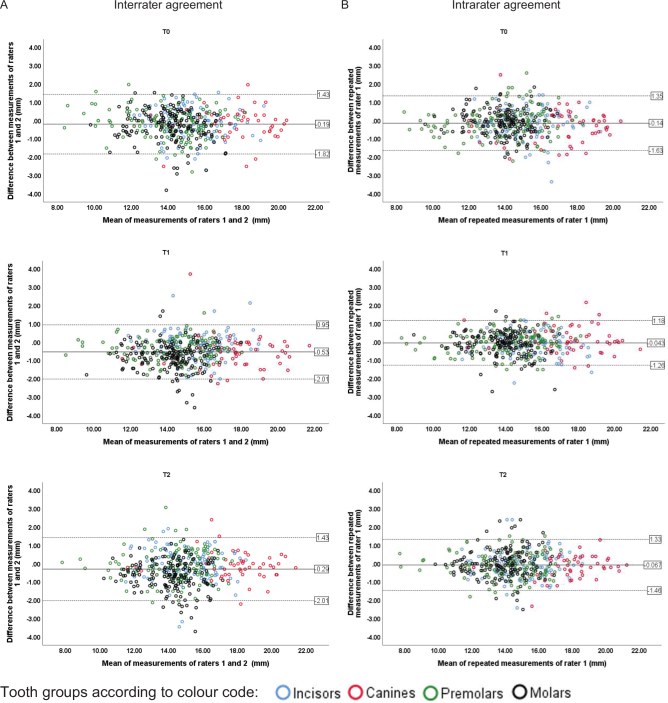
Agreement between repeated measurements of root length by Bland–Altman plots illustrating the interrater (A) and intrarater (B) agreement (in millimetres). Upper and lower 95% limits of agreement (±1.96 SD) varied between +1.43 mm and −2.01 mm for different tooth groups and timepoints. Therefore, within these limits, 95% of the mean value differences between repeated measurements were found. The colour-coded markers represent roots of the four different tooth groups. SD: standard deviation; T0: time before treatment; T1: time during treatment; T2: time after treatment.

Upper and lower 95% limits of inter- and intrarater agreement varied between +1.43 mm and −2.01 mm for different tooth groups and timepoints (Figure 3).

**Figure 4:**
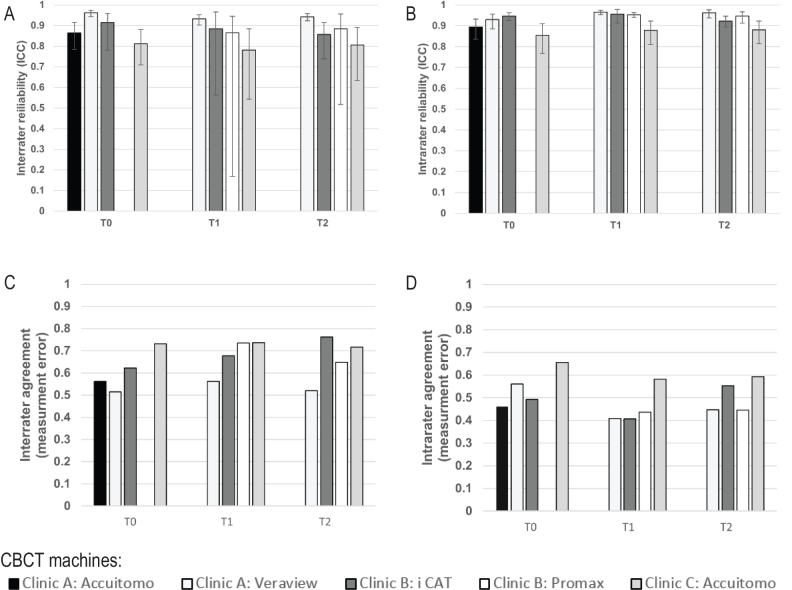
Comparison of repeated measurements of root length between images from the five different CBCT machines. Inter- and intrarater reliability estimated by intraclass correlation ranged between 0.78 and 0.97 (95% CI: 0.19–0.98) (A, B) and agreement estimated by measurement error (Dahlberg’s formula) ranged between 0.41 and 0.76 (C, D). CBCT: cone beam computed tomography; T0: time before treatment; T1: time during treatment; T2: time after treatment; CI: confidence interval.

Only minor differences were shown regarding reliability and agreement comparing CBCT examinations performed on different CBCT machines at the three different radiological clinics (Figure 4).

## Discussion

This study successfully included two raters for measurement of root length using MPR in CBCT volumes, resulting in good to excellent reliability, and measurement error for agreement of 0.41 to 0.77 mm, confirming our hypothesis. This indicates that the measurement method has good reproducibility, and thus good quality. Hence, this study adds new knowledge concerning the measurement process in whole. Also, the measurement error estimated in this study may be valuable in future research projects as well as in clinical settings where EARR is estimated to determine the validity of root lengths measurements [11].

Reliability and agreement have various definitions in the literature and are often used interchangeably which can be somewhat confusing. In the current study, we used the concepts as recommended in GRRAS [11]. Also, several different methods have been used for the statistical analysis of reliability and agreement whereof a combination of coefficients is recommended [10, 11]. Hence, in the current study, ICC was used to assess reliability, whereas Dahlberg measurement error and Bland–Altman plots [22] were used for assessment of agreement [11]. Not only statistical decisions are needed to interpret study results and to evaluate the method´s usefulness, but also an assessment of the intended clinical application of the measurements is crucial [10, 11]. The results should be interpreted in the context of clinical relevance, but there seems to be no consensus regarding what is considered clinically relevant in terms of root shortening, although a cut-off level of 2.0 mm has been suggested [5, 9]. This is consistent with the level of severe root-shortening according to the index for visual assessment of EARR in intraoral radiographs by Malmgren [24]. This value conforms with our results of the Bland-Altman plot with upper and lower 95% limits of agreement varying between +1.43 mm and −2.01 mm for different tooth groups and timepoints, meaning that only root length changes exceeding 2.0 mm can be considered valid.

The reliability and agreement are not only dependent on the imaging technique but also on the raters’ different cognitive, visual and perceptual abilities [25]. Therefore, it is a strength in our study that the two raters had different experience: one is an orthodontist and the other is an oral and maxillofacial radiologist. Ideally, more than two raters are recommended to ensure that the measurement method is reproducible [12]. For research concerning root length measurements in CBCT, this study nevertheless adds essential knowledge, as few studies have analysed reliability and agreement with more than one rater. Furthermore, as the measurements had good to excellent reliability and the agreement was judged to be good, it was deemed that no additional rater was needed. Training and calibrating raters in reliability and agreement studies is crucial [26], allowing for the control of systematic errors to achieve reliable and consistent results. The benefits of a calibration process may diminish at long intervals between the measurements. Nevertheless, avoiding the risk of recall bias is important; therefore, the repeated measurements interval in our study was a minimum of 4 weeks. Considering the large number of roots measured, the risk of recall bias would likely have remained minimal, even with shorter intervals. However, there seems to be no consensus regarding the optimal length of intervals for repeated measurements in this type of study. While random errors can be mitigated by increasing the sample size, they are challenging to completely eliminate due to various factors, including differences in the cognitive, visual, and perceptual abilities of the raters, which can result in varying assessments and measurements [12, 26].

There were factors that the observers subjectively experienced as challenging, some of which may have affected reliability and agreement. In earlier studies, the reference points for root length measurement have been the apex and CEJ [14, 27], or the cusp/incisal edge [28]. As the cusp/incisal edge may change over time due to attrition, it is preferable to use the CEJ as the reference point, as it is more stable when comparing root lengths from different occasions. However, using the CEJ as a reference point is challenging, as this point is located at different levels with different degrees of visibility in the reconstructed images using MPR. This especially applies for displaced and rotated teeth, lower incisors, and molars [29]. Additionally, the visibility of the CEJ can be obstructed by material artefacts from dental restorations; but in the current sample, no exclusions were needed for that reason.

In the current study using MPR, the three reference planes (axial, sagittal, coronal) were oriented according to the length axis of each individual root. This posed a risk that the reference planes were different in the remeasurements by the same or the second rater. Therefore, this study of inter and intra-reliability was important as its results will constitute the basis for the clinical RCT-study where the prevalence of external root resorption (EARR) will be evaluated. Also, a displaced tooth at baseline (T0) may indicate a higher risk of EARR. Accordingly, the measurements from the three different timepoints were assessed independently to minimise the risk of recall bias in the measurements at T1 and T2.

For the study results to be clinically applicable, root length measurements were made at three timepoints: before (T0), during (T1) and after treatment (T2), as recommended in clinical praxis. At the timepoint during treatment, metal brackets were bonded to the teeth, posing a potential risk of decreased image quality and interpretability due to metal artefacts [30]. The result from the study by Lund et al. showed the lowest intrarater reliability at the timepoint with metal brackets on the teeth being explained by the ongoing remodelling of the apical part of the root, which made it difficult to identify [14]. The results of the current study showed a bit higher interrater measurement error for canines at T1 compared with T0 and T2, which could be explained by how the tooth development of the canines was ongoing during the study. Hence, the apex was open, making it indistinct and hard to identify. In addition, the canine roots were often curved and sometimes had to be measured in two steps. Nonetheless, the level of interrater and intrarater reliability and agreement comparing different timepoints and tooth groups was relatively high and even (Figure 2). Interrater measurement error for molars was slightly higher and ICC slightly lower with wider CI compared to other tooth groups, which is not surprising given that molars were the tooth group that we found to be the most difficult to assess during the calibration process. This may be because the complex anatomy of multirooted teeth makes correct positioning of the root difficult during MPR.

Although the CBCT parameters were calibrated according to image quality in relation to radiation dose and adapted to the different CBCT machines, our subjective opinion is that the image quality varied between different CBCT machines. The objective analysis, however, showed only minor differences in the reliability and agreement, implying that using different CBCT machines did not affect the results (Figure 4).

The current study constitutes a pre study of an RCT aiming to evaluate root length changes and the worthiness of intermediate radiography. It is a well-known fact that the use of CBCT involves increased radiation dose compared to intraoral radiography. To achieve the best possible evidence, CBCT was nevertheless used since it has been shown to be superior to intraoral radiography in the evaluation of root length for research purpose [13, 27].

The results show high and relatively even reliability and high agreement for the two raters across different timepoints, tooth groups and CBCT machines, indicating high reproducibility and quality of the measurement method using MPR in CBCT. Compared to the only previous study that utilised MPR in CBCT to measure root length for assessing EARR [14], the current study had several advantages. Firstly, the current study involved two raters, enabling assessment of interrater reliability. Secondly, roots of 336 teeth were assessed in this study, compared to 156 in the previous study [14], enabling increased robustness of the results. Finally, the current study used different CBCT machines, thus increasing knowledge concerning reliability and agreement in multicentre studies.

### Future research

To increase the validity of clinical and radiological studies, simultaneous assessments of reliability and agreement are essential. Further, development of artificial intelligence-driven measurements that can potentially minimise human error could enhance the quality of assessments. In the current study, root length measurements were opted to make comparison with other studies possible. In future studies we intend to contribute to the development of root volume assessments using the CBCT images obtained in the RCT.

## Conclusions

CBCT measurements of root length on images from different machines during different phases of orthodontic treatment had good to excellent reliability and agreement. Measured values for root shortening with MPR in CBCT that are less than 2.0 mm are uncertain, as they may contain measurement errors. Measurements of root shortening exceeding a clinically relevant level of 2.0 mm are valid for research and clinical purposes.
